# Philadelphia Academy of Surgery

**Published:** 1894-04

**Authors:** 


### *SOCIETY REPORTS.*

---

### PHILADELPHIA ACADEMY OF SURGERY.

PHILADELPHIA, February 5, 1894.

DR. OSCAR H. ALLIS read the following paper, entitled, " A Further Communication upon the Use of Carbolic Acid" :

One of the results of my article on "Carbolic Acid," read before this Society in October last, was the reception of two practical and interesting letters upon the same subject from physicians who were no strangers to the valuable services attainable by the proper use of the drug. By permission of the writers I am permitted to present their views before the Society.

The first is from Dr. B. H. Brodnax, Brodnax, La. He writes:

"In 1868 I came near shooting off my right wrist. The explosion of the gun was so near that it set fire to my shirt. I was therefore suffering not only from a severe traumatism, but also from a painful burn. Dr. Hart, of Pickins, Miss., dressed the wounds and burns with a mixture containing ten per cent. of carbolic acid in linseed oil. The pain ceased, and I slept well all night. Dr. Hart remarked as he dressed my wounds, 'You are the first man in Mississippi to have a wound dressed with carbolic acid.' This was twenty-five years ago, and during that time I have never found a drug to take its place. Other so-called 'antiseptics' have been given a trial, and that is all.

"No part of a physician's experience is more valuable than when he is the sufferer ; and you will pardon me if I add one other personal experience. In 1880 while soldering a broken tin pipe, I accidentally spilled some molten solder on my hand between the thumb and first finger. I flirted off the mass, about the size of a silver dollar, which took the skin with it and caused most intense suffering. Near at hand stood a bottle of carbolic acid. I took the bottle, and, with the cork wet with the drug, smeared the fresh wound. This was too slow business, so I inverted the bottle and poured some of the acid into the wound. As if by magic all pain disappeared, the burning and smarting stopped, leaving a sense of refreshing coolness. What surprised me quite as much as anything else was my ability to finish the work that I had begun with as much ease and unconcern as if nothing had happened. The cure, too, was prompt and uneventful.

"My next case was a woman seventy-five years of age. She had burned the upper surface of the foot so severely by upsetting scalding water, that when the stocking was removed the cuticle came with it. The poor woman was in great agony when I arrived. The raw surface was fully eight inches long by five inches wide. I took a feather which I had picked up as I entered the yard, and with it spread the undiluted acid over the entire raw surface, and extending the application over the parts adjoining and which were less deeply burned. In a few seconds all pain ceased, and the patient remarked, 'It feels as if cold water was being poured over it.'

"The third application was to a child three years old who, while at the breakfast table, pulled the coffee-pot over, emptying the contents into its bosom. Its clothing was removed leaving the neck and breast of the child denuded. I immediately applied the officinal strength of carbolic acid to the raw surface, covering also the unbroken blebs with the same. Pain ceased almost instantly, when the child permitted me to cut the blebs and paint them without a whimper.

"My after-dressing to the burnt surface is very simple. I apply directly to the raw surface absorbent cotton, which I retain in place with a bandage. This is the first and usually the only dressing, for when it comes off the part is healed.

"As my practice has been of a general character, I have not had a large number of any one class of cases, but think that I have used the carbolic acid in at least twenty burns and scalds. Two more cases that I recall may interest you. A man was burned by a jet of steam that skinned the forearm from the elbow to the wrist. The carbolic acid was applied, and with the same result, the man remarking, 'I thought I was in for two weeks, but I'll be at work again in the morning.'

"A child, one year old, got a live coal in the bottom of its shoe. The burned area was about the size of a five-cent piece, and deep. The child was nearly frantic with pain. I dipped a small piece of cotton into the pure acid and pressed it into the burned hole. I *counted* eighteen seconds, when the child stopped screaming, and, looking up at me said, 'It done ; it has stopped hurting.'

"I always keep the pure crystals on hand ; and to them add, when I require their use, just enough water to dissolve them, possibly ten per cent. This is strong enough to remove the the skin from healthy parts. I do not add the water to dilute the acid. When it is deliquesced, as it sometimes is, I use it without any water.

"While, probably, carbolic acid has no rival in its curative action upon burns and scalds, its medicinal influence does not stop here. In sciatica I have found that the hypodermic injection of from five to ten drops of a solution of twenty drops of carbolic acid to the ounce of water carried down to the seat of pain produces almost instant relief. So, too, in carbuncles and boils, I take a solution of from twenty-five to fifty drops to the ounce of water and inject deeply into the inflamed area a couple of drops in three or four places. On the following day the injection is repeated if necessary. The inflamed area is covered with a little absorbent cotton soaked in :

R Carbolic acid ............................................gtt. xxx.

Tannin.......................................................ʒ iv.

Aquæ.........................................................℥ ij.

"By this means the morbid process is arrested and resolution promptly takes place.

"I treat gonorrhea with injections of the strength of ten drops of carbolic acid to the ounce of water, giving also *per orem* two or three drops three times a day. Cure usually in about three weeks.

"I have never but once used it upon a freshly-cut surgical wound, as Dr. Gardner suggests. In this case I removed a urethral caruncle of large size, and which was an obstacle to micturition. After clipping off the growth with scissors taken from the lady's workbox, I immediately applied undilute acid to the cut surface. The pain, smarting and hemorrhage ceased promptly."

In regard to the use of the deliquesced carbolic acid in burns, Dr. Gardner confirms what Dr. Brodnax claims for it. He says:

"While the Alabama and Chattanooga Railroad was in process of construction, from 1869 to 1874, I, as surgeon to the road, had between five and six thousand men under my care. Among the injuries burns and scalds were frequent and often serious. These would vary in size and depth, sometimes involving the extremities and a large portion of the trunk. One foreman, in particular, had a large part of the back and a lesser amount of the front portion of the trunk, with portions of the legs, seriously burned. On removing the clothing, large portions of the skin came off with it. His condition was lamentable in the highest degree. Morphine was freely administered internally, while to the entire raw surface carbolic acid was applied. On the bed upon which he was to recline, cloths soaked in carbolized oil were laid. Where the blisters were unbroken the carbolic acid was applied with a view of toughening the skin. Where blebs were present these were treated to a thin coating of the pure acid and let alone. The relief was very great. It is the only treatment that I have ever used in the burns and scalds."

The following is from the pen of Dr. C. J. Cleborne, Medical Director of the United States Naval Hospital, Chelsea, Mass. He writes:

"I believe I was the first to use carbolic acid in full strength (Merck's crystals) in buboes, boils, carbuncles and glandular swellings generally, and can add confirmatory experience of twenty-five years to that of Dr. Gardner's. I made free openings in suppurating buboes, squeezed out the pus, applied the liquefied acid to every part, dressed it with zinc ointment, and often in a fortnight the sailor was discharged to duty.

"On one occasion my apothecary on shipboard, who was greatly enthusiastic over the curative effects of carbolic acid upon buboes, injected, upon his own responsibility, nearly a drachm of the melted crystals into the urethra of a marine. *That gonorrhea ceased then and there,* but that apothecary did not show himself to the marine for a week, for there was murder in his eye! No stricture or other evil followed, but I should not fancy it would be the favorite injection. I have often used it as an analgesic, painting it over a felon before cutting. Dr. Ashhurst, I think, has used alcohol on cut surfaces after operations; but I think Dr. Gardner must be the first to use *carbolic alcohol*—for that is all it is—so extensively.

"Carbolic acid in a dilute form is readily absorbed. I have detected hydrochianine in the urine of women using carbolic soap in vaginal injections, the urine turning very dark in some cases; but in its full strength I do not think it is ever absorbed. I have used it in hydrocele and in cysts about the neck and jaws, and in no instance have I noticed constitutional effects. The prompt albuminizing of tissues with which the acid comes in contact renders them incapable of absorption. I regard it as the mildest and most superficial of all escharotics. I have known carbolized oil to prevent the healing of ulcers and wounds. In such instances I believe it cuts down the healthy delicate granulations, acting as an irritant. It is often useful as an application in chancroids and venereal herpes. I prefer it to the tincture of iodine as an injection into small cysts. I have injected two drachms into cysts about the jaws. In such, any excess may be slowly absorbed, but I have never known ill effects from its use."

I wish to call special attention to the use of liquid carbolic acid, full strength, or, if the crystals are used, only enough water added to make a solution, as an immediate application to all varieties of burns. Neither Dr. Brodnax nor Dr. Gardner claims originality* in the use of the drug, but to my mind it is one of the boldest ventures in all surgery. To think of applying to a raw and agonizing burn that which would scald a healthy cutaneous surface would seem to the unreflective mind a reckless and wantonly cruel act. But when we consider that a raw burned surface is painful from its exposed nerve-filaments, and that the strong acid combining with the albumen of the tissues forms a coating that excludes the air, while at the same time it benumbs—paralyzes—each terminal exposed nerve-filament, the remedy seems to be the result of a happy inspiration.

*Dr. Charles W. Dulles, author of "What to do First in Emergencies," and who is familiar with the literature upon the treatment of burns, says that he has never seen any mention of the use of the undilute acid as a topical remedy.

The lesson to be learned also from the injection of a drachm of the liquefied crystals into the urethra as mentioned by Dr. Cleborne proves that if any necrotic action is ever present, that action must be very superfiicial, for in the case referred to there were no immediate or ulterior bad results.

I do not care to add to the contribution of Drs. Brodnax and Cleborne, and will close by urging a careful use of the drug in deep sinuses, no matter in what direction they run nor how deep they may penetrate. I am now using it in a case of spinal caries in which the catheter passes in six or eight inches before it stops. This I have injected several times, with a great relief of pain and amelioration of every symptom.

DISCUSSION.

DR. W. W. KEEN: I would ask Dr. Allis if there is not danger of carbolic acid poisoning from the use of the drug; and also in regard to sloughing of the tissues. We have all seen much sloughing take place around hemorrhoids in consequence of the injection of carbolic acid into the body of the hemorrhoid.

DR. DEFOREST WILLARD: Within a week I have seen a case where pure carbolic acid had been injected into a hemorrhoid. The entire lower part of the rectum, the anus, the perineum, scrotum, and penis were involved in a large slough from which the patient speedily died.

DR. ALLIS, in concluding the discussion, said that from the tenor of the remarks of some of the Fellows of the Academy it was probable that they were not present when the former paper was read. For the benefit of such he would briefly say that Dr. Gardner, of Bloomsburg, had been in the habit of using carbolic acid in full strength upon the entire surface of flaps after amputation, and over the cut surface after removal of the female breast, and that union *per primam* resulted. Such cases fully established the principle claimed, that the strong acid combines with the fresh tissues forming a protective albuminate, a condition which renders further absorption impossible. The same takes place when the strong acid is applied to a raw burned surface. It is not claimed that an aqueous dilutation is safe when applied extensively to raw surfaces; on the contrary, the more dilute the more dangerous. Dr. Mears tells of a case in which bloody urine was passed by a person who had been present when the carbolic spray was used in an operation. Here systemic effects were produced by a spray so dilute that it did not irritate the lungs. In a case of washing out the thorax in the treatment of purulent pleurisy, the late Roger Keys, a most careful and judicious physician, came near losing a patient from absorption of the dilute acid. It will strike many of you with astonsihment when I say that it would be safer to pour a gallon of pure carbolic acid into a purulent thoracic cavity than to pour in a gallon of water into which a single ounce of carbolic acid had been placed. I will even go further and say, that excess of the strong acid in a cavity such as an abscess cavity, or upon exposed tissues as a burn or a fresh wound, does no harm, while excess of a dilute solution if left in a cavity, or used over an extensive raw surface, will be promptly followed by dangerous if not fatal toxic effect.

The diverse results obtained from the hypodermic use of the strong acid contain an important lesson. It has been said that Dr. Levis in his use of carbolic acid in hydrocele had cases of extensive sloughing of the scrotum; these were, however, rare; but Dr. Levis had also very many cases in which a similar use of the acid was followed with the happiest results. So also in the case just mentioned of extensive sloughing of the anus and perineum from injections of carbolic acid in hemorrhoids; while this is true, it is also true that the same acid has been used safely in thousands of cases of hemorrhoids. Besides, too, the most extensive sloughing of the lower end of the rectum that I have ever known could not be traced to any cause. While therefore every one must acknowledge that carbolic acid may do great mischief when misapplied, the experience of Gardner, Brodnax and Cleborne demonstrate beyond cavil that we have in it an agent of incalculable good, and one that the profession will do well to cultivate.

